# Recent Advances in Sugarcane Genomics, Physiology, and Phenomics for Superior Agronomic Traits

**DOI:** 10.3389/fgene.2022.854936

**Published:** 2022-08-03

**Authors:** Mintu Ram Meena, Chinnaswamy Appunu, R. Arun Kumar, R. Manimekalai, S. Vasantha, Gopalareddy Krishnappa, Ravinder Kumar, S. K. Pandey, G. Hemaprabha

**Affiliations:** ^1^ Regional Centre, ICAR-Sugarcane Breeding Institute, Karnal, India; ^2^ ICAR-Sugarcane Breeding Institute, Coimbatore, India

**Keywords:** superior agronomic traits, genomic estimated breeding value, genomic selection, next-generation sequencing, high-throughput phenomics

## Abstract

Advances in sugarcane breeding have contributed significantly to improvements in agronomic traits and crop yield. However, the growing global demand for sugar and biofuel in the context of climate change requires further improvements in cane and sugar yields. Attempts to achieve the desired rates of genetic gain in sugarcane by conventional breeding means are difficult as many agronomic traits are genetically complex and polygenic, with each gene exerting small effects. Unlike those of many other crops, the sugarcane genome is highly heterozygous due to its autopolyploid nature, which further hinders the development of a comprehensive genetic map. Despite these limitations, many superior agronomic traits/genes for higher cane yield, sugar production, and disease/pest resistance have been identified through the mapping of quantitative trait loci, genome-wide association studies, and transcriptome approaches. Improvements in traits controlled by one or two loci are relatively easy to achieve; however, this is not the case for traits governed by many genes. Many desirable phenotypic traits are controlled by quantitative trait nucleotides (QTNs) with small and variable effects. Assembling these desired QTNs by conventional breeding methods is time consuming and inefficient due to genetic drift. However, recent developments in genomics selection (GS) have allowed sugarcane researchers to select and accumulate desirable alleles imparting superior traits as GS is based on genomic estimated breeding values, which substantially increases the selection efficiency and genetic gain in sugarcane breeding programs. Next-generation sequencing techniques coupled with genome-editing technologies have provided new vistas in harnessing the sugarcane genome to look for desirable agronomic traits such as erect canopy, leaf angle, prolonged greening, high biomass, deep root system, and the non-flowering nature of the crop. Many desirable cane-yielding traits, such as single cane weight, numbers of tillers, numbers of millable canes, as well as cane quality traits, such as sucrose and sugar yield, have been explored using these recent biotechnological tools. This review will focus on the recent advances in sugarcane genomics related to genetic gain and the identification of favorable alleles for superior agronomic traits for further utilization in sugarcane breeding programs.

## 1 Introduction

Sugarcane (*Saccharum* spp.) is a major industrial crop grown in tropical and subtropical regions across the world. It is cultivated mainly for sugar production and supplies >70% of the world’s sugar. Additionally, it also provides a sustainable and renewable source of bioenergy. It is cultivated in over 24.9 million hectares in about 80 countries with a production value of 174 million tons (OECD-FAO Agricultural Outlook report 2019–2020). India is the world’s second largest sugar producer (26.6 million tons) after Brazil (40% of world production). China, Thailand, the United States, Pakistan, Mexico, and Russia are the other major sugar-producing countries in the world. In India, substantial sugar production is projected for the coming years as a result of higher cultivation rates of promising sugarcane varieties and good rainfall during the 2020 season (OECD/FAO, 2021).

The modern cultivars of sugarcane originated from an interspecific hybridization between *S. officinarum* (2*n =* 8*x =* 80; *x =* 10) and *S. spontaneum* (2*n =* 5*x–*16*x =* 40–128; *x =* 8). Around 70–80% of the genome is from *S. officinarum*, while 10–20% is from *S. spontaneum*; the remaining 10% of the genome has arisen from interspecific recombination (D’Hont et al., 1998; Piperidis and D’Hont, 2020; Garsmeur et al., 2018). The sugarcane genome is larger (about 10 GB) than any other member of the Poaceae family (Aitken et al., 2016) and is highly aneuploid, polyploid, and heterozygous in nature. This is because most cultivars of sugarcane have 110–130 chromosomes. The monoploid genome (800–900 Mb) is larger than that of sorghum (790 Mb); recently sequenced by the French Agricultural Research Centre for International Development (CIRAD) using the R570 cultivar (Garsmeur et al., 2018).

It is hoped that progresses in genome sequencing and the accumulation of genomic resources such as the SUEST (sugarcane expressed sequenced tag) database, genetic maps, transcriptomes, bacterial artificial chromosome (BAC) libraries (Souza et al., 2011), etc. will help in understanding the evolution of the sugarcane’s complex genome and polyploid structure. Complete genome sequencing will also help to unravel key genes/pathways associated with superior agronomic traits in sugarcane. Although substantial genetic enhancement for cane and sugar yield in modern cultivars has been achieved through conventional breeding approaches, the rate of genetic gain in sugarcane crops is comparatively slower than those of other cereal crops (Yadav et al., 2020). Breeding varieties with superior agronomic traits is highly desirable for sustained sugarcane production under changing climate scenarios across the word.

Genomic selection (GS), which utilizes genome-wide genetic markers to estimate breeding value, when used along with genomic strategies, can substantially increase selection efficiency and genetic gain in sugarcane breeding programs. Furthermore, selection intensities in sugarcane breeding programs can be further enhanced by utilizing large breeding lines and high-throughput phenotyping and genotyping with bioinformatics tools.

In the wake of enormous advancements in sugarcane genomics, we outline in this article the recent progress and future possibilities in sugarcane genomics and phenomics in identifying superior alleles for agronomically superior traits in sugarcane crops. These desirable/superior alleles/traits can be incorporated into sugarcane breeding lines either to improve existing cultivars or to create new cultivars with enhanced cane and sugar yield. These approaches can ensure sustainable increases in sugarcane production with available resources to meet growing demands.

### 1.1 Role of Next-Generation Sequencing in Genomic Selection

The size of the sugarcane genome is ∼10 Gb, which is approximately three times that of the human genome (Le Cunff et al., 2008; Souza et al., 2011; Zhang et al., 2012). Genome size analyses of sugarcane varieties using flow cytometry have revealed that the sugarcane genome may be as large as 3.80–10.96 Gb (Berkman et al., 2014). Chromosomal characterizations of different sugarcane clones via genomic *in-situ* hybridization (GISH) have revealed that clones with 80 chromosomes are classic *S. officinarum*, while those with more than 80 chromosomes are inter-specific hybrids between *S. spontaneum* and *S. officinarum* (X. Liu et al., 2018). The development of a correct sequence assemblage has been hindered by the absence of diploid progenitors. Furthermore, the construction of a reference genome for an inter-specific sugarcane hybrid having both homo and homoeologous chromosomes is a difficult task (Souza et al., 2011).

Sorghum is considered to be the reference model for comparative studies due to it being closest in synteny with sugarcane (Le Cunff et al., 2008; de Setta et al., 2014; Aono et al., 2021). Next-generation sequencing (NGS)-based genotyping is now popularly used in many crop species that lack reference genomes and has become the primary choice for many breeders due to its cost-effectiveness and speed (Chung et al., 2017). NGS technology has enriched our understanding of the complex sugarcane genome and its evolution (Zhang et al., 2018). The availability of a draft genome sequence, along with 76 K single nucleotide polymorphisms (SNPs) (Yang et al., 2017), a further 84 K SNPs (Balsalobre et al., 2017), and the Axiom Sugarcane100 K SNP array (You et al., 2019) will pave the way for the implementation of genomic selection (GS) in commercial sugarcane breeding programs.

Genomic selection is a powerful tool for increasing genetic gain rates and shortening breeding cycle lengths (Meuwissen and Goddard, 2001; Crossa et al., 2017). It can open new avenues for sugarcane breeders in managing breeding programs precisely and in more cost-effective ways. Sugarcane breeders need not wait till the harvest stage or maintain entire breeding populations to screen for traits of interest during phenotypic evaluations at different crop stages. This can reduce the time and cost of selection processes. Based on genomic estimated breeding values (GEBVs), a sugarcane breeder can select desired clones predicted to have superior phenotypes. By adopting this approach, a breeder can estimate the potential breeding values of the desired clones before introducing them into main breeding programs. The individual clonal entries are first phenotyped for desired traits and then genotyped using genome-wide marker resources available for sugarcane; these results are then further used to predict the breeding value of clones using prediction models. Thus, GEBVs predicted from reference breeding populations and clones with high genetic gain or breeding value can be easily identified at the earlier stages of the breeding cycle. This approach was first applied in animal breeding programs, reducing the animal breeding cycle by up to 5 years. GS has been successfully implemented in many crops, such as maize, rice, sorghum, and wheat (Daetwyler et al., 2012; Gaffney et al., 2015; Spindel et al., 2015; Fernandes et al., 2018), where several quantitative traits have been accurately predicted using GS. However, there are few studies that utilize this approach for sugarcane.

In commercial crops like sugarcane, GS has tremendous potential to achieve genetic gains for major desirable yielding traits, such as cane yield, number of millable canes (NMC), uniform tillering population, single cane weight (SCW), high biomass, commercial cane sucrose (CCS), etc., which are polygenic with each gene exerting small effects. It is challenging to improve such traits through marker-assisted selection (MAS) approaches (Gouy et al., 2013). Using GS is more advantageous than pedigree-based methods of breeding or MAS, since pedigree-based methods are slower and MAS fails to address loci with complex traits governed by many genes with small effects (Heffner et al., 2009). As GS can capture both large and small quantitative trait locus (QTL) effects, complex traits including cane yield and quality-based traits can be predicted more effectively and accurately in shorter periods of time (Crossa et al., 2017).

### 1.2 Genomic Selection Models

Several predictive models have been developed for GS studies in crop plants; however, selecting the correct model is crucial for high prediction accuracy. In general, the prediction accuracy of GS models varies according to their assumptions and the treatment of marker effects (Liu et al., 2018). In crop plant GS studies, genomic best linear unbiased prediction (G-BLUP) and ridge regression best linear unbiased prediction (RR-BLUP) are commonly employed prediction models. In RR-BLUP, all markers are assumed to have equal variances and small but non-zero effects, although this assumption does not imply that the effects of all markers are equal (Bernardo and Yu, 2007). RR-BLUP provides high prediction accuracy when the trait is controlled by several loci with small effects (Buckler et al., 2009). G-BLUP is another widely used model that uses genome-wide markers to predict the genetic and phenotypic values of selection candidates (Wang et al., 2015). The common assumption in G-BLUP and RR-BLUP models is that the effects of all loci have a common variance, making them more suitable for use in predicting traits influenced by a large number of minor genes. Most markers across whole genomes produce small or no effects, whereas a few markers produce huge effects according to G-BLUP and RR-BLUP assumptions.

Most Bayesian methods, however, allow different markers to have different effects and variances, unlike G-BLUP and RR-BLUP. As part of the BayesB model, most loci are assumed to have no effect on a trait, and thus most markers are excluded from the prediction model. If the trait expression is governed by large-effect QTLs, which explain much of the genetic variability, BayesB fits well (Munkvold et al., 2009). The parameter in BayesCπ; however, π can be calculated by using experimental data, through which the shrinkage degree is determined, and therefore, this model is more feasible than BayesB for real data analysis. The BayesA model is more suitable for traits governed by a moderate number of genes because its shrinkage degree is lower than BayesB and BayesCπ. Generally, Bayesian methods result in better predictions due to their ability to capture large-effect QTLs. Bayesian least absolute shrinkage and selection operator (LASSO) combines the features of subset selection with the shrinkage produced by Bayesian regression. The reproducing kernel Hilbert space (RKHS) method combines an additive genetic model with a kernel function and converts predictor variables into a set of distances among observations to produce a definite matrix that can be used in a linear model (Gianola et al., 2006).

Selective shrinkage models, such as BayesB, BayesCπ, BayesA, and Bayesian LASSO, are sensitive to a number of QTLs; as the numbers of QTLs increase, prediction accuracies decrease (Wang et al., 2015). In contrast, the prediction accuracy of G-BLUP and RR-BLUP often stay nearly constant regardless of the numbers of QTLs and are more suitable for traits governed by a large number of minor genes.

### 1.3 Training Population and Breeding Population

Sugarcane breeding programs aim to improve genetic gains for major traits such as cane and sugar yield. However, there is negative correlation between commercial cane sugar and cane yield, indicating that many of the genes controlling these traits are independent. Genetic gain for these traits through phenotypic selection is very low. This is because the accumulation of superior alleles for these traits via phenotypic breeding value is very rare (Jackson, 2005; Mahadevaiah et al., 2021). Therefore, it is only possible to accumulate favorable alleles for these traits through recurrent selection schemes (Lingle et al., 2010). GS using recurrent selection methods has shown enhanced genetic gain and improvements in prediction accuracies for many desired traits in sugarcane (Yadav et al., 2020). Combining GS with recurrent selection-derived breeding populations and multi-environment phenotyping of breeding can help in increasing prediction accuracies and genetic gains for superior agronomical traits.

Under present sugarcane breeding programs, phenotypic selection takes 12–13 years to develop a new variety for commercial cultivation. The crosses effected once are maintained in a clonal form and evaluated over the breeding cycle. Hence, chances of introgression of new alleles through recombination are possible only at the beginning of the crossing program, which is carried out in once every 12 years. Furthermore, handling a large number of seedlings in the ground nursery and carrying them forwards in the subsequent stages of first and second clonal trials before entering pre-zonal variety testing is a herculean task (Meena et al., 2020a). These tasks are time consuming, labor intensive, and expensive because they require the maintenance of clonal lines in large land areas. GS can help in reducing the numbers of clonal lines that need to be maintained in every stage of the breeding program. Since it also utilizes additive and non-additive variance components in genomic prediction models, the technique can help in integrating many desirable alleles into selected clonal lines (Deomano et al., 2020; Voss-Fels et al., 2021). Once the desired traits are accurately phenotyped in multi-environmental locations via recurrent selection methods, genomic prediction can also help in identifying desirable selections from the target breeding pool for optimal performance in various agro-ecological regions.

Therefore, GS can aid in rapid genetic gains in sugarcane breeding through the selection of superior clonal entries at the early crop stage; this helps in reducing the length of the breeding cycles in such programs.

### 1.4 Genomic Selection in Sugarcane Breeding

**FIGURE 1:**
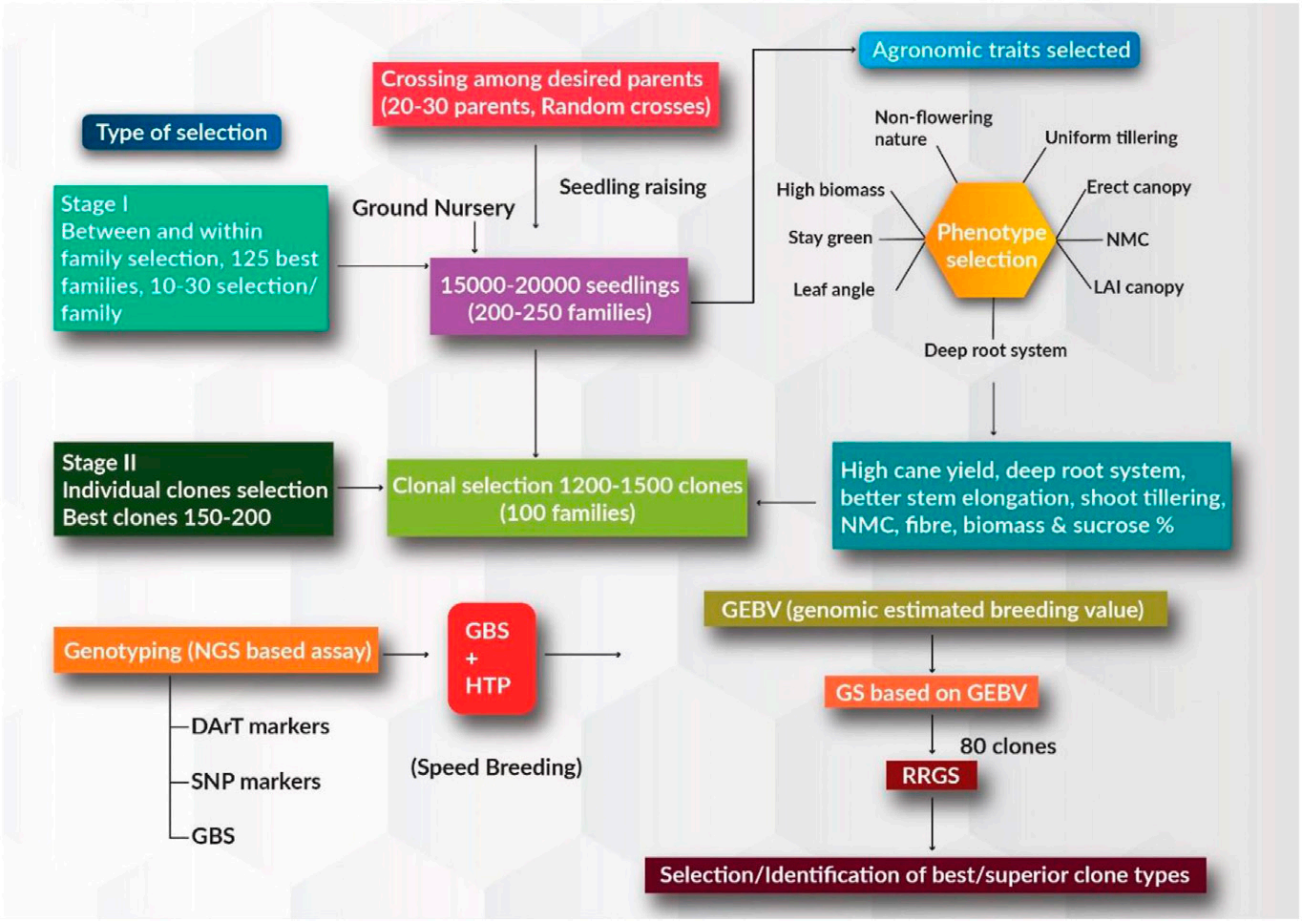
Schematic for genomic selection (GS) approach in sugarcane for increasing the rate of genetic gain and reducing generation intervals. Stage 1 represents the training population where phenotypic selection is carried out based on desirable major agronomical traits. Stage 2 represents individual clonal selection where the best clones are selected, and genomic estimated breeding value (GEBV) estimation is performed.

Continuous advances in plant breeding and agronomic practices in the context of systematic crop improvement have contributed significantly to annual productivity gains in major food commercial crops. However, the demands for food, sugar, fiber, energy, and fuel are growing due to the ever-increasing human population, increasing per capita income, and diversified food consumption/industrial use patterns. Although sugarcane has been cultivated primarily as a source of sugar for centuries, the crop has recently gained much attention as a source of ethanol and biodiesel to satisfy global energy demands. Owing to its economic value, the sugarcane genome has received growing interest in recent times in the context of the changing climate. In the current breeding program, ground nursery populations (∼15–25 K), generated through the random crossing of desired parents (∼20–30 parents) in the national hybridization garden facility, can be evaluated for desired agronomically superior traits. Within and between family selection is employed and around 100–125 improved families can be selected from a population of 15–25 K seedlings (training population) based on the desired trait value. Individual selection is employed at the first and second clonal stages based on increasing cane and sugar yielding traits, better stem elongation, shoot tillering, NMC, deep root system, and high biomass production, depending upon the breeding objective. GEBVs for these desired traits can be estimated using SNP markers in the first and second clonal stages (breeding population) (Figure 1). Prediction accuracies for these superior traits based on genome-wide marker information and phenotypic accounts are expected to be high. Best performing clones with superior agronomical traits can be used as parents for further rapid recurrent GS to accumulate the favorable alleles for these desired traits. Better performing clones with high phenotypic trait values are selected using rapid recurrent GS approaches; these clones can either be used as parents in ongoing breeding programs or be evaluated with existing varieties in order to identify better varieties in zonal varietal trials.

Recent advancements in sugarcane genomics, including the compilation of a draft genome sequence, are opening new windows in the utilization of genomic resources for rapid genetic gain through molecular breeding tools such as genome-wide association studies (GWAS) and GS. In GS, available genome-wide markers are used to estimate the effects of all loci and thereby calculate the GEBV, which has a substantial potential for increasing selection efficiency. GS in livestock breeding has incredible potential in accelerating genetic gains (sometimes as high as two-fold) compared to conventional progeny testing (Hickey et al., 2017; Georges et al., 2019). Therefore, GS and GEBV likely hold great potential to accelerate the rate of genetic gain in sugarcane breeding (Yadav et al., 2020).

In any sugarcane breeding program, high cane yield and CCS yield are two major traits of commercial importance, and they are governed by many genes and/or QTLs (Aitken et al., 2008). Cane yield has high narrow-sense heritability and a non-additive nature of genetic variance (Wei and Jackson, 2017). Recent advancements in genomic prediction and the availability of diversity array technology (DArT), Affymetrix Axiom 100 K SNP arrays, and Axion 345 K SNP arrays (Aitken et al., 2016; You et al., 2018) could provide ample opportunities to speed up genetic gains for many agronomically superior traits. These include cane yield, sucrose content, NMC, SCW, erect canopy, leaf angle, non-flowering nature, high biomass, and deep root system, and they can be selected for through the accurate prediction of breeding values in candidate parent clones and the shortening of generation intervals. The selection of parents based solely on breeding values in conventional breeding may not improve the genetic gain for cane yield due to low narrow-sense heritability and very high non-additive genetic variance (Wei and Jackson, 2017). In a simulation study of sugarcane for genetic gain (Voss-Fels et al., 2021), two different GS-based breeding strategies were compared with standard pedigree selection (PS) over five breeding cycles. GS Scheme 1 was similar to conventional PS, along with three rapid recurrent genomic selection (RRGS) steps. In contrast, GS Scheme 2 was fundamentally different from PS as it did not include a progeny assessment stage, but did include three RRGS steps. Both GS models achieved annual genetic gains of 2.6–2.7%, which were 1.9 times higher than those of normal PS with an additive model (1.4%). In non-additive models, annual rates of genetic gain were lower for both the schemes; however, the rates for the GS schemes (1.5–1.6%) were still higher than those of PS (1.1%). Similarly, GS was used by Islam et al. (2021) to test for two major diseases in sugarcane—brown and orange rust—and the authors observed that GS prediction accuracies ranged from 0.28–0.43 and 0.13–0.29, respectively. The prediction accuracies further improved when a known major gene for brown rust resistance was included as a fixed effect in the GS model. Genome-wide markers such as SNPs have also been utilized for genomic prediction studies in sugarcane (Gouy et al., 2013; Deomano et al., 2020; Hayes et al., 2021; Yadav et al., 2021). The results of these studies provide further motivation for adopting GS-based techniques in sugarcane breeding programs. The different factors affecting GS accuracy are genetic relatedness, type and density of molecular markers, heritability of the trait, gene effects (additive/non-additive/interactions), population size, population structure, statistical models used to calibrate the best-fitted model, extent and distribution of linkage disequilibrium (LD) between markers and genomic regions associated with the trait of interest, effective population size, and genotypes–environment interactions (GEI) (Meuwissen and Goddard, 2001; Zhang et al., 2017; Berro et al., 2019).

### 1.5 Genomic Selection for Superior Agronomic Traits

The development of breeding sugarcane clones with agronomically superior traits is slow and expensive. During recent decades, the rates of genetic improvement in sugarcane have been static compared to other crops (Wei and Jackson, 2017). Therefore, the use of modern approaches, such as speed breeding, GS, and genome editing, could accelerate the accumulation of favorable alleles for several desired agronomic traits. Many traits of agronomically superior value, such as uniform tillering habits, erect canopy, non-flowering nature of the plant, high sucrose yield, deep root system, ability to stay green, high NMC, high SCW, higher cane yield, high commercial cane sugar production in tonnes per hectare (CCS t/ha), and high biomass and fiber production, are considered to be important traits in the main sugarcane breeding programs (Meena et al., 2020b) (Figure 1). The combinations of these traits further depend upon the breeding objective of the researchers. The main objective of commercial breeding is to develop good clones/varieties with improved performance. If the breeding objective is for a high yielding energy crop, then clones with higher biomass and fiber, profuse tillering, and high biotic/abiotic stress tolerance are selected. Similarly, if the objective is to develop clones for high cane and sugar yield, then agronomically desired traits such as long internodes, high stalk weight and numbers, and higher sucrose production for more commercial cane sugar are considered. Further, if the development of clones suitable for drought stress is the main objective, desired traits such as deep root systems, erect canopies, and staying green, along with high cane and sugar yield are given importance. Likewise, to obtain clones with good ratooning ability, traits such as higher ratooning potential during the winter and spring season are given more focus. Combining these traits based only on breeding values may not be adequate to obtain genetically superior individuals. This is because only substantial amounts of additive genetic variance of these traits of interest are captured; however, the overall performance of clones is determined both by additive and no-additive genetic variance. Hence, breeding approaches that exploit both these genetic components together need to be given priority. The inclusion of non-additive effects in genomic prediction models improves clonal prediction accuracies significantly, which can further enable the precise identification of sugarcane clones/varieties (Yadav et al., 2021).

Therefore, the successful application of GS approaches for these important traits could substantially accelerate genetic gain and increase the accuracy of the selection of desired traits in sugarcane breeding.

### 1.6 Effect of Markers and Prediction Accuracies

As GS is an innovative approach in sugarcane crop, few researchers have reported the accuracy of GS approaches for economically important traits in sugarcane. GS in sugarcane was first reported by Gouy et al. (2013), who found small to substantial levels (0.11–0.62) of genomic prediction accuracies for 10 agronomically important traits using a small panel of 167 individuals with 1,499 DArT markers. The lesser prediction accuracy in this study could be due to the small population size and few marker numbers used. Recent assessments of GS in sugarcane, with modestly sized reference populations, have shown more promising prediction accuracies for key commercial traits (Gouy et al., 2013; Deomano et al., 2020). There are many factors, such as trait heritability, training population size, marker density, and statistical models, that influence genomic prediction accuracies in sugarcane (Heslot et al., 2013). The extended statistical models that combine the effects of genome-wide markers with non-additive effects could further improve genetic gains in clonal selection programs (Yadav et al., 2020). However, the crucial elements determining the prediction accuracies of models are dependent on the genetic architecture of the desired trait, along with type of gene action governed. Additive genetic variance is a proportion of genetic variance inherited in progeny, which is helpful in the selection of desired parental combinations. Non-additive variance, on the other hand, is helpful in the selection of desired plant types during selection from the ground nursery (Jackson et al., 2016; Mahadevaiah et al., 2021). As cane yield is reflected by low narrow-sense heritability and CCS has a moderate level of heritability, also governed polygenically (Hoang et al., 2015), both non-additive and additive effects must be taken into account to improve the overall performance of these traits.

In another study by Yadav et al., (2020), the genomic prediction accuracies for cane, CCS, and fiber yields using G-BLUP, extended G-BLUP, and RKHS models were compared. The study found that GS-based extended G-BLUP improved prediction accuracies significantly. With the available genome-wide SNP marker arrays and statistical prediction models, the exploration of non-additive gene action for many desired complex traits to accelerate genetic gain in sugarcane breeding can be undertaken (Yadav et al., 2021). Although there has been tremendous progress in NGS and genotyping technology, the variable ploidy levels and multi-allelic dosage effects in sugarcane still pose a big challenge in breeding (Piperidis and D’Hont, 2020). Therefore, considerations of allele dosage effects in genomic prediction are needed for more accuracy in assessing genotypic effects (de C. Lara et al., 2019; Endelman et al., 2018). To overcome such challenges, single dose markers (SDMs), which follow segregation patterns similar to those of diploid species and pseudo-diploid models, remain the key options for linkage analysis and GS in sugarcane breeding. Under pseudo-diploid models, heterozygous genotypes are considered in one class in genotype calling (Deomano et al., 2020; Yadav et al., 2021); further details of this technique in sugarcane breeding can be found in Aitken et al. (2016).

Recent progress in GS techniques in sugarcane for key cane traits, such as cane yield, sugar yield, fiber percent, and other traits, has been found to provide good prediction accuracies (Deomano et al., 2020; Hayes et al., 2021; Yadav et al., 2021). GEBV accuracies for flowering traits, such as days to flowering, and pollen and gender viability with high heritability of 0.57, 0.78, and 0.72, respectively, were assessed using three genomic prediction approaches, namely G-BLUP, BayesR, and genomic single step (GenomicsSS) (Hayes et al., 2021). The prediction accuracy for many of the traits (0.3–0.44) indicated that GS could be used to improve these traits in sugarcane breeding programs. The genomic prediction accuracies for sugar and cane yield (0.25 and 0.45) were found to be promising in an Australian breeding program by Deomano et al. (2020). In this study, the researchers used three relatively large commercial sugarcane varieties (2,400 clones) with a 47–57 K SNP array. In another study, a total of 1,276 sets of parental clones with their progenies were used with a 22 K genome-wide SNPs marker array to predict GEBV, which was then correlated with phenotypic breeding value (Ansari et al., 2020). The identified correlations for major breeding traits, such as sucrose (0.45) and cane yield (0.60), further support the use of GS to predict the breeding value for the subsequent selection of superior cross combinations.

### 1.7 Physiological Approaches for Superior Agronomic Traits

Sugarcane is also valued for other products besides sugar. The population density of a sugarcane crop has a strong influence on the agronomic output *viz*. the production of high sucrose, fiber, biomass, bioenergy, ethanol, etc. The population density is characterized by the numbers of canes, leaf area, biomass, and canopy structure for efficient solar energy harvest for the photosynthetic conversion of light energy into carbohydrates. Cane yield also depends on several plant characteristics, and the two factors collectively enhance yield per unit area.

#### 1.7.1 Optimum Shoot and Cane Population

Tillering or underground branching is responsible for the crop stands in sugarcane. Tillering, in general, peaks at 120 days after planting (DAP) and declines thereafter due to mortality, and usually stabilizes at 180 DAP. Thereafter, the majority of shoots are converted to cane stacks (Vasantha et al., 2012). Inter- and intra-row spacing influences shoot/cane population density, SCW, and cane yield. Hence, working out optimum spacing for higher yield is imperative to match with resource limitations for specific growing conditions.

#### 1.7.2 Physiological Traits Contributing to Yield

Considerable knowledge has been gained concerning the physiology of yield in crops and it is now possible to quantitatively estimate limits to crop yield based on physiological and environmental constraints (Sinclair, 1993; Sinclair, 1994; Muchow et al., 1997).The two main components of sucrose yield are biomass and sucrose fraction, and increasing one or both of these will increase sucrose yield. Maximizing radiation interception, its conversion and partitioning to stalk (Singles and Donaldsons, 2000), and the amount of photosynthetically active radiation (PAR) intercepted by a canopy determines the biomass produced. Genotypes exhibit variations in canopy development and structure (Zhou, 2003). Source leaf photosynthetic capacity is correlated with decreases in assimilate availabilities to the acropetal sink tissue (McCormick et al., 2006). Inman-Bamber et al. (2009) are of the opinion that contrasts in sucrose content reside more in the morphology of the plant and responses to ripening stimuli, such as mild water stress, and how these traits influence supply and demand for photo assimilates. Therefore, in the early stages, sugarcane crop selection should focus on yield alone, while the focus on sucrose content should be stronger in later stages of varietal evolution (Zhou, 2004). Inman-Bamber (1991), Inman-Bamber (1994), and Zhou (2003) determined the patterns of tiller populations among sugarcane genotypes. The harvest index (HI) has been overlooked or misunderstood in sugarcane as it is unique among field crops as the economic product is also in the harvested stalk; therefore, special considerations are required for assessing the importance of HI (Moore, 1989; Evans and Lloyd, 1993).

Ideotypes of crop plants based on canopy temperature depression (CTD) has been proposed by Blum (2005) for use in plant breeding according to drought types, such as isohydric (“water saving”) and anisohydric (“water spending”) models. The water saving model has a distinct advantage in harsher environments, whereas the water spending model is expected to perform relatively better under more moderate/mild drought situations. Sugarcane hybrids with high water use efficiency (WUE) can play a pivotal role in sustaining sugarcane productivity by reducing the volume of irrigation water required for its cultivation in water-scarce regions of India. The deficit irrigation treatments (50% by volume and 50% by frequency) lead to declines in cane yield of 41.2 and 56.4%, respectively. WP has been found to be significantly influenced by irrigation level; the reduction in irrigation water reduced WP by 17.5 and 36.3% in restricted irrigation treatments (Tayade et al., 2020). Significant reductions in CTD, the ratio of variable fluorescence to maximum chlorophyll fluorescence (*Fv*/*Fm*), the soil plant analytical division (SPAD) index, and the leaf rolling index were observed under limited irrigation during the grand growth stage of sugarcane (Arun kumar et al., 2020). This study highlights the significance of CTD and *Fv*/*Fm* as useful physiological tools for selecting sugarcane clones suitable for production under water-limited conditions. Several physiological traits, such as chlorophyll fluorescence, CTD, SPAD index, photosynthesis, and transpiration efficiency (Luo et al., 2013; Jackson et al., 2016; Liu et al., 2019), are related to photosynthetic efficiency and total biomass. In sugarcane, traits associated with biomass tend to have positive relations with cane yields and yield components. Canopy-based physiological parameters need to be assessed for their influence on cane yield specifically in pre-breeding and advanced breeder trials. Tiller and leaf development parameters and biomass partitioning are potential yield predictors. The heritability of the most promising parameter should be studied to exploit the potential of the crop. Mamet and Galwey (1999) suggested that shoot elongation parameters are reliable indicators of the time of ripening. Singh and Venkatarama (1983) reported that early ripening sugarcane cultivars had higher growth rates than others during elongation, which lends weight to the idea that early varieties are physiologically more efficient since the early varieties were taller as well as faster growing than the late varieties. In sugarcane, changes to plant architecture and crop geometry are being explored to breed crops better suited to mechanical harvest, intercropping, modified fertigation methods, and crop intensification. The concept of ideotypes has thus been modified; hence, studies on these aspects must also be undertaken.

**FIGURE 2:**
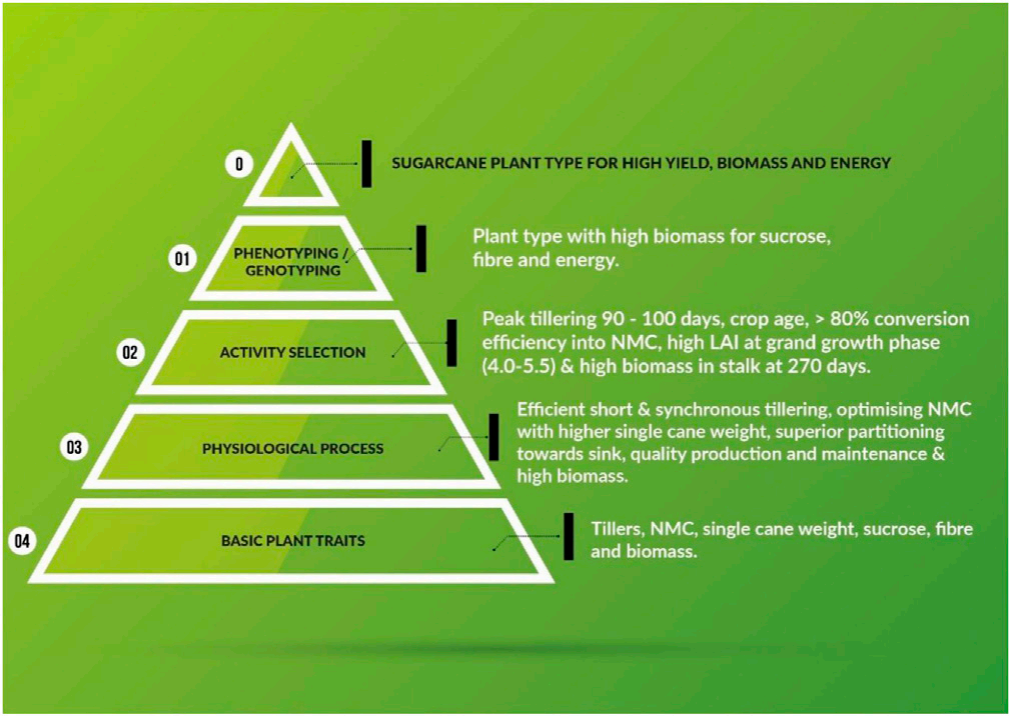
Schematic illustrating primary plant traits of sugarcane that increase efficiencies in physiological and economic output.

The major aim of plant breeding science is to develop plant types with enhanced plant traits and physiological processes. The schematic representation of the traits of primary nature, which improve physiological and economic output efficiencies is depicted in Figure 2. The bottom tier of the triangle represents the total numbers of tillers (ultimately resulting in NMC), NMC (actual harvestable part of the crop), SCW (a major component of cane yield), sucrose (measurable quality of the produce), and biomass (efficiency of solar energy harvested and converted to chemical energy). All the traits presented in the first tier are measurable primary data. The second tier lists desirable physiological activities culminating in better output of the processes, *viz*. early synchronized tillering, conversion efficiency of tillers to canes, optimized NMC for higher CCS%, superior partitioning towards economic traits, and the maintenance of high quality and high biomass for varied end products (sucrose, bioenergy, and biofuel). The quantifiable and desirable physiological plant behaviors for efficient output are depicted in tier three. The penultimate tier explains both phenotyping and genotyping for the aforementioned physiological and plant processes. Most of these processes have been defined by specific phenotype and genotype markers by sugarcane researchers (Moore and Botha, 2014). The ultimate outcome, i.e. the ideal sugarcane plant, should also meet the demands of the sugar industry.

### 1.8 High-Throughput Phenomics

Using non-invasive methods (such as sophisticated cameras combined with automation) can help in studying complex phenotypes in sugarcane crops rapidly. The usefulness of unmanned aerial vehicles (UAVs) has been clearly reviewed by Cardoso et al. (2022) for its usefulness in precision agriculture for the cultivation of sugarcane in Brazil, a crop with the third largest planted area in the country. High-throughput phenotyping also aids in predicting complex traits that are appropriate for selection and provides relevant information on why particular genotypes perform well under specific conditions (Pasala and Pandey, 2020). Arun kumar et al. (2020) reviewed studies on shoot/root growth, plant architecture, greenness, leaf area, leaf rolling, leaf angle, leaf curvature, leaf senescence, growth rates, tillering, early vigor, plant height, phenology, biomass, convex hull, compactness, and eccentricity, all of which could be studied easily with the aid of cameras (Golzarian et al., 2011; Das et al., 2016). Infra-red cameras used for thermal imaging have revolutionized screening methods for drought-tolerant genotypes in wheat, rice, and other crops. Such cameras, used in conjunction with UAVs or drones, can be used to identify drought-tolerant sugarcane clones in available pools of germplasm. Thermal imaging can be helpful in understanding stomatal conductance and plant health both under biotic and abiotic stresses (Moller et al., 2006; Talamond et al., 2015). The usefulness of UAV-assisted canopy temperature (Tc) measurements in sugarcane has clearly been emphasized (Basnayake et al., 2016) compared to that of the old-style, lengthy, and labor-intensive measurement through handheld infrared cameras. Arun Kumar et al. (2020) recently highlighted that canopy temperature depression (CTD) in sugarcane clones grown under water-limited conditions had a significant positive correlation with cane yield.

Near infrared imaging and multispectral line scanning cameras can be useful for determining water content, leaf thickness, leaf area index (Zhang et al., 2021), and root soil moisture extraction patterns. Hyperspectral cameras can also be useful in studying several physiological traits, *viz*. chlorophyll content, relative water content, nutrient status, chemical composition, plant health, and photochemical reflectance index (Römer et al., 2012; Sahoo et al., 2015; Arun kumar et al., 2016; Wahabzada et al., 2016). Field phenomics that encompass the measurement of phenotypes occurring both in cultivated and natural conditions are much more useful in sugarcane crop breeding studies than controlled environment phenomics research, which involves the use of glass houses, growth chambers, and other systems.

Cane height is highly influenced by the soil, total sugar content, leaf nitrogen content, temperature, and light intensity, and it can be an indicator of yield and other parameters (Souza et al., 2017). Bunruang (2021) showed a significant correlation (*R* ^2^ = 0.82) between measured plant height (PH) and estimated PH, reflecting the usefulness of aerial images from a UAV three months prior to harvesting.

#### 1.8.1 High-Throughput Phenomics for Early-Stage Selection

The effectiveness of a high-throughput phenotyping system was demonstrated by Natarajan et al. (2019); it can be adapted to capture and use indirect yield-related traits for clone selection from early-stage selection trials in sugarcane breeding programs. Ground observations using various sensors, *viz*. visual, multispectral, and thermal mounted on UAVs in six-month-old sugarcane plants, revealed that stalk number and plant height were highly correlated to canopy cover (rg = 0.72) and canopy height (rg = 0.69), respectively (Natarajan et al., 2019). They also reported that UAV-assisted high-throughput phenotyping is considered an imperative strategy for improving clonal selections and genetic gains in early-stage sugarcane breeding programs.

#### 1.8.2 Phenomics for Pre-Harvest Cane Yield Determination

Recently, Som-ard et al. (2018) reported a successful (more than 90% accuracy) forecasting model for pre-harvest sugarcane yield determination using UAV-acquired RGB color images coupled with ground information data. The high spatial resolution of the UAV image and the advanced image classification of object-based image analysis (OBIA) with a gray-level co-occurrence matrix demonstrated the significant potential for the prediction of sugarcane yield before harvest, which will be beneficial to sugarcane farmers and related industries. Further adding to this discussion, Tanut et al. (2021) reported a new robust tool, *viz*. the wonder cane model, which correctly forecast total sugarcane harvest yield with an accuracy of 98.69% with fewer estimate errors. The wonder cane model determines the cane yield based on the principle of through data in classification form rather than data in regression form.

#### 1.8.3 Big Data Management in Plant Phenomics

Phenomics generates a hefty number of images and metadata through phenotyping instruments, hence there is a prerequisite for proper data processing and management (Kim et al., 2017). High-throughput phenomics (HTP) has helped researchers to record the crop data in a faster way, both in a temporal and spatial manner. However, in HTP, the big data (data = phenotypic data + molecular data + environmental data) (Rahaman et al., 2015) generated over the period remains a huge task, and recently many articles have addressed this issue of big data management. The large set of images and the meta-data generated automatically can be properly processed, standardized, and stored, and finally combined with genomic models. Proper big data management will render HTP successful in crop breeding programs for increased crop productivity under the changing climate. Reliable, automatic, and high-throughput phenotyping platforms have been developed to solve this complex problem of big data management in plant phenomics.

The large set of germplasm in various crops, and the high-throughput phenotyping along with the intelligent management of big data through proper phenomic platforms *viz.* PHENOPSIS, WIWAM, PHENOSCOPE, GROWSCREEN, TraitMill, PHENODYN, PlantScan, LemnaTec, Qubit Phenomics, and HRPF (Rahaman et al., 2015) helps in identifying the outlier, and finally reduction in background noise, leading to the proper utilization of data correlated with the required component, *viz.* crop yield and biomass. The implementation of proper statistical tools in data analysis, *viz.* PCA, factor analysis, discriminant analysis and stepwise regression, linear mixed model, generalized linear model (GLM), likelihood estimation of mixed models, multiple regression, and artificial neural network (ANN), favors the accelerated selection of better genotypes in various crops against abiotic and biotic stresses under the changing climate. Tests for multicollinearity, autocorrelation, and heterogeneity will be extremely useful in solving the complex data analysis (Rahaman et al., 2015).

The correct identification of background noise, for example in thermal imaging for canopy temperature measurement the air temperature (Tair), net isothermal radiation (Rni), vapor pressure deficit (VPD), and wind speed, multiplies the background noise in canopy temperature determination, and the dissection of leaf temperature needs stable weather conditions (Costa et al., 2013).

Thorough understanding of the sugarcane phenotype according to the phenophase (Vasantha et al., 2021) [i.e. germination phase (0–60 days): nodal roots and other root formation along with canopy coverage/vigor; Formative phase (60–150 days): tiller production; grand growth phase (150–240 days): optimum leaf area index; maturity and ripening phase (240–360 days): SCW, cane thickness, cane length, invertase, SPS, and SS activity] will be extremely valuable in assimilating the complex pathways and physiological and biochemical traits, ultimately leading to better association with crop biomass and yield. A multidisciplinary team consisting of experts in data acquisition, image processing, machine learning algorithms, computer applications, and statistics helps in rendering the HTP for improved sugarcane phenotyping in meaningful way.

### 1.9 Genome Editing in Sugarcane

The recent assembly of a draft sugarcane genome has accelerated crop improvement. In spite of this, its polyploid nature, the large size of its genome, low transformation efficiency, and lack of high-throughput tools and transgene silencing in sugarcane poses a great challenge for these efforts (Souza et al., 2011; Mohan, 2016; Okura et al., 2016). The genome-edited plants with site directed nucleases have a competitive edge over the transgenic plants in terms of biosafety regulations. In sugarcane producing countries, such as Brazil and India, the edited plants with site directed nuclease (SDN1 and SDN2) approaches are considered non-transgenic or foreign-DNA-free. SDNs have increased the possibilities worldwide for researchers and breeders to alter the genomes of target organisms in a way that is extremely difficult or impossible to achieve using conventional breeding (Shan et al., 2013).

Among the highlights of sugarcane genome editing are the generation of events incorporating and expressing TALEN, capillary electrophoresis experiments to demonstrate targeted mutation, analysis of 89–148 nucleotide PCR amplicons encompassing the TALEN target site, and phenotypes that show reduced lignin and altered lignin and cell wall composition under greenhouse conditions (Jung and Altpeter, 2016; Meena et al., 2020b). Sun et al. (2016) utilized TALEN scaffolds and the transformation system to achieve targeted caffeic acid O-methyltransferase (COMT) mutation at an efficiency of 74%, which is comparable to previous studies that have reported mutation rates of between 4 and 31%. A field evaluation was carried out by Kannan et al. (2018) to determine the agronomic performance, cell wall composition, lignin content, as well as the saccharification efficiency of these TALEN-mediated COMT mutant sugarcane lines. By demonstrating TALEN-mediated targeted mutagenesis in sugarcane, a new option for genetic improvement has opened up despite its complex polyploid genome. To create a “loss of function” phenotype in sugarcane, extensive co-editing of many targets is necessary. These authors found that lignin biosynthesis gene COMT co-editing more than 100 copies/alleles did not have any adverse effects on performance under field conditions. Targeted mutagenesis of the more than 100 COMT copies/alleles led to a decrease in the amount of lignin and a change in the ratio of lignin monomers. This was in addition to improving the saccharification of cell wall-bound sugar by 39–44%.

#### 1.9.1 The CRISPR-Cas System (Clustered Regularly Interspaced Short Palindrome Repeat)

This genome-editing based on RNA-guided nucleases has emerged as a one of the most simple and versatile methods (Gasiunas et al., 2012; Chandrasegaran and Carroll, 2016; Oz et al., 2021) during the past decade. The CRISPR-Cas mechanism involves the cleavage of DNA through specific nucleases, for example, CRISPR-Cas9, and repair by non-homologous end joining (NHEJ) or homology-directed repair (HDR), to rectify double-strand breaks (DSBs). Non-homologous end joining results in knockout alleles through frameshift mutations due to the loss of nucleotides, while HDR leads to the introduction of precise genetic modifications, including single-nucleotide substitutions, gene replacements, and large insertions (Huang and Puchta, 2019). The CRISPR-Cas9 system has been successfully employed to modify cereal, vegetable, and horticultural plant species to improve their agronomic traits (Xu et al., 2019; Ansari et al., 2020; Tian, et al., 2021). The challenges in sugarcane, being of a polyploid nature with a large number of homeologs and homologs, could have functional redundancy of alleles. Nevertheless, the CRISPR-Cas9 system offers an opportunity to generate the efficient multiallelic co-editing of a large number of copies of genes. Higher efficiency of editing can be achieved through optimization of the genome-editing reagents and their delivery to enable the efficient co-editing of a large number of copies/alleles (Eid et al., 2021).

Genome-edited varieties with improved agronomic traits are becoming a reality in sugarcane using this technology. Brazil has developed non-transgenic CRISPR-edited sugarcane, Cana Flex I and Cana Flex II, with modified cell wall components and high sucrose content, respectively. Cana Flex I plants facilitate ethanol production and better extraction of other bioproducts. Eid et al. (2021) used the CRISPR system to target the multiallelic magnesium chelatase subunit I (*MgCh*) gene, which is a key gene in chlorophyll biosynthesis. The CRISPR-Cas9 system could target 49 copies/alleles of magnesium chelatase, as confirmed via Sanger sequencing. The edited plants produced light green to yellow leaves. This work proved that genome editing could be achievable in polyploid crops, overcoming the limitations of the protospacer adjacent motif site (Eid et al., 2021).

The CRISPR-Cas system has been successfully used to produce herbicide tolerance in sugarcane (Oz et al., 2021). The co-editing of multiple alleles of the acetolactate synthase gene was done through a template-mediated HDR approach. This technology can be used in both directions, i.e. either elimination of inferior alleles or random addition of desirable alleles. The alleles of a susceptible variety can be eliminated to make it resistant and *vice-versa*. In future, many agronomically desired traits, such as an increase in chlorophyll content to enhance the photosynthesis and reduce the nitrogen requirement, herbicide resistance to advance weed management, and an increase of multigene to improve biomass yield in sugarcane (essential to acquire more biofuel from plant sources to replace the dependency on petrochemicals and save the environment), can be improved in the existing cultivars.

As far as the regulatory aspects of genome editing are concerned, the process is different for different countries. In the US, gene-editing events without transgenes do not require regulatory approval by the US regulatory agencies. In Brazil, the National Biosafety Technical Commission (CTNBio) states that the classification can be made on a case-by-case analysis by CTNBio for the varieties of sugarcane. Cana Flex I and II are classified as non-transgenic. In India, the Department of Biotechnology, Ministry of Science and Technology, and the Department of Agriculture Research and Education, Ministry of Agriculture and Farmers Welfare have recommended that the SDN1 and SDN2 genome-edited products that are transgene-free should be exempt from biosafety assessment.

### 1.10 The Role of Artificial Intelligence and Machine Learning in Big Data Analysis

Data-intensive modes of research, both in basic and applied plant sciences, have gained momentum over the last two decades due to greater advances in the field of big data generation (Leonelli et al., 2017). Advances in high-throughput genomic platforms (HTGPs), coupled with public genomic data sharing and high-throughput phenotypic platforms (HTPPs), has significantly increased the capacity to dissect biological complexity more efficiently (Tardieu et al., 2017). High-throughput phenotyping thorough non-invasive imaging techniques, including thermal, digital, and spectroscopic imaging, as well as chlorophyll fluorescence, are more useful in extracting the information, which helps in the biological elucidation of plant growth (Walter et al., 2010; Chen et al., 2015). Both plant phenotyping and genotyping have improved progressively over the recent past, and big biological data generation through HTGPs and HTPPs has contributed to a boom in artificial intelligence (AI) and machine learning (ML) in commercial agriculture to deliver precision farming strategies.

The simulation of human intelligence in machines that are programmed to think like humans and mimic their actions is generally refereed as the AI. It refers to machines displaying characteristics of the human mind (Frankenfield, 2021). Machine learning (ML) is a sub-domain of AI where the machine can learn automatically from data without being explicitly programmed. AI methods require large data repositories in order to effectively program machines for desired tasks. To enhance early detection and thereby improve decision-making, AI algorithms require big data sets for training (Frey, 2018). The data generated through non-destructive phenomics using instruments and sensors, such as digital cameras and spectrometers, are generally integrated with their own proprietary communication protocols into AI algorithms. It is often necessary to convert sensor-based data to digital formats before analyzing it (White et al., 2012). In order to utilize phenomics data management, Artificial Intelligence (AI) is mainly used for: the conversion of sensory data into phenotypic information; the development of models to comprehend genotype-phenotype relationships with environmental interactions; and the management of databases to facilitate the sharing of information and resources (Pauli, 2015). The main aspects of AI, ML, deep learning, and computer vision have been applied thus far to a recognizable extent in phenomics. Similarly, high-throughput NGS technologies have been emerging to generate economically viable big data sequence sets to address the continuous demand for cost-effective sequence data generation.

The recent advancements in the field of AI have presented ample opportunities; AI applications in crop research and agriculture have so far primarily benefited large-scale industrial farming (Carbonell, 2016), with research and development investment mainly focused on: commodity crops, such as wheat, rice, and maize; high-value horticulture crops, such as soft fruits; and the enhancement of large-scale orchards and vineyards. Yield forecasting in agricultural crops is a challenging task; to address this, Murali et al. (2020) developed a ML hybrid model with available data for yield forecasting. Combining a statistical model, such as generalized autoregressive conditional heteroscedasticity (GARCH), with a recurrent neural network (RNN) refined using the whale optimization algorithm, was found to be more appropriate for sugarcane yield forecasting in the medium term. Similarly, Kumar et al. (2015) used ANN-based models for the prediction of sugarcane yield in India. The ML algorithm has also been used to predict the sugarcane yield by using the support vector machine (SVM) algorithm (Kudagi et al., 2020). In a similar attempt, Rodrigues and Pereira (2021), applied ML to normalized difference vegetation index (NDVI) images to estimate sugarcane productivity. ANN has also been used in the individual selection process within the best sugarcane families to increase the sugarcane breeding efficiency; ANN made the same selective choice as the breeder during the simulation for the individual best linear unbiased predictors (BLUPs) (Brasileiro et al., 2015).

A novel deep learning (DL) approach with three different extractor models (VGG-16, VGG-19, and Inception v3) and seven (naive Bayes, AdaBoost, neural network, stochastic gradient descent, K-nearest neighbor, support vector machine, and logistic regression) different classifiers has been used to classify sugarcane crops as diseased and non-diseased with the highest area under curve (AUC) of 90.2% (Srivastava et al., 2020). In a similar attempt, AI has been used to detect sugarcane plant disease by using the discrete wavelet transform (DWT) algorithm (Reddy et al., 2017). The fast and precise detection of stem nodes on sugarcane crops in the complex natural environment is a pre-requisite for the development of sugarcane harvesters. An object detection algorithm based on deep learning was used by Chen et al. (2021) for stem node recognition in the sugarcane field conditions. The four different deep learning algorithms (Faster R-CNN, SSD300, RetinaNet, and YOLO v3) were compared with the YOLO v4 algorithm, and the average precision of the sugarcane stem node detection by YOLO v4 was 95.17%, which was superior compared to other tested algorithms. Zhou et al. (2020) used a new sugarcane seed cutting system based on machine vision to save time and labor intensity. The different segments of the sugarcane stalks were identified by the machine vision with a recognition rate of 93% and an average time of 0.539 s, along with zero rate of bud damage. Sugarcane is a long duration crop, and during its course of growth and development, a lot of weed infestation poses a threat to sugarcane yield. In this context, Hashemi-Beni et al. (2022) used deep learning to improve weed control and precision agriculture through the fast processing of big datasets captured by unmanned aerial systems (UASs) for smart and precision agriculture. These authors fine-tuned U-Net to classify sugarcane ortho-mosaic UAS datasets into three classes (background, crops, and weeds) based on its performance on SegNet, FCN-32, FCN-16, and FCN-8 deep learning models to distinguish crops from weeds (Hashemi-Beni et al., 2022).

## 2 Conclusion

The integration of GS techniques in current breeding programs will be very useful in identifying better-performing sugarcane clones at earlier stages than conventional breeding techniques. GS can not only help in reducing population sizes but can also shorten breeding cycles and help in the selection of parents with superior agronomic traits. Utilizing rapid recurrent selection in superior clones can help in accumulating desired alleles associated with superior agronomic traits, such as cane yield, sucrose percent, NMC, uniform tillering, staying green, erect canopy, non-flowering habit, deep root system, better stem elongation, and tolerance to biotic and abiotic stresses. Identifying clones with high accuracy helps in increasing genetic gains for complex traits in sugarcane breeding programs. Implementing GS techniques with modified breeding schemes can have great potential to increase selection efficiencies and the rate of genetic gain in sugarcane. Real-time monitoring of phenomic changes with respect to the response of plants to different stresses has been simplified by the genomics revolution, which has alleviated the bottlenecks in phenomics. Automated and remote sensors, data integration, and experimental design all contribute to effective phenotyping by increasing the accuracy, precision, and throughput of measurements, as well as reducing costs due to rapidly evolving low-cost phenotyping facilities. The invention of chemicals and physical mutagens has shown a multifold improvement in creating genetic changes that have shaped a new revolution in agriculture production. The introduction of the precise and specific biologically highly efficient tool CRISPR-Cas9 is expected to accelerate the beneficial genetic change in crop plants, assisting in feeding the world’s growing population. Advancement in the application of AI and ML is expected to significantly improve instant planning and decision making and maintain product quality.
